# Association of prognostic nutritional index with long-term survival in lung cancer receiving immune checkpoint inhibitors: A meta-analysis

**DOI:** 10.1097/MD.0000000000041087

**Published:** 2024-12-27

**Authors:** Lei Wang, Xingxia Long, Ying Zhu, Ailin Luo, Mei Yang

**Affiliations:** a Department of Thoracic Surgery, West China Hospital, Sichuan University, Chengdu, China; b West China School of Nursing, Sichuan University, Chengdu, China.

**Keywords:** immune checkpoint inhibitor, lung cancer, meta-analysis, prognostic nutritional index, survival

## Abstract

**Background:**

This meta-analysis aimed to identify the association of prognostic nutritional index (PNI) with long-term survival in lung cancer patients who received the immune checkpoint inhibitors.

**Methods:**

The Medline, CNKI, EMBASE, and Web of Science databases were searched up to August 20, 2023. Progression-free survival (PFS) and overall survival (OS) were main outcomes and hazard ratios (HRs) and 95% confidence intervals were combined. Subgroup analysis stratified by the pathological type [non-small cell lung cancer (NSCLC) vs small cell lung cancer (SCLC)], previous treatment history and combination of other treatment was performed.

**Results:**

Twenty-two available studies with 2550 patients were included. Pooled results demonstrated that lower PNI was related to worse PFS (HR = 0.51, *P*<.001) and OS (HR = 0.43, *P*<.001). Furthermore, subgroup analysis based on the pathological type (non-small cell lung cancer: HR = 0.52, *P*<.001 for PFS, HR = 0.41, *P*<.001 for OS; small cell lung cancer: HR = 0.41, *P* = .007 for PFS, HR = 0.44, *P* = .007 for OS), previous treatment history (first-line treatment: HR = 0.67, *P*<.001 for PFS, HR = 0.52, *P*<.001 for OS) and combination of other treatment (No: HR = 0.54, *P*<.001 for PFS, HR = 0.43, *P*<.001 for OS; Yes: HR = 0.63, *P*<.001 for PFS, HR = 0.51, *P*<.001) showed similar findings.

**Conclusion:**

PNI is significantly associated with long-term survival in immune checkpoint inhibitors treated lung cancer and patients with lower PNI are more likely to experience poorer prognosis.

## 1. Introduction

Lung cancer is the most common malignant tumor in China and globally, with types including non-small cell lung cancer (NSCLC) and small cell lung cancer (SCLC). Unfortunately, most lung cancer patients are diagnosed at an advanced stage.^[1,2]^ In addition to conventional treatments such as surgery, chemotherapy, and radiotherapy, immunotherapy, particularly immune checkpoint inhibitors (ICIs), has emerged as a new option for the treatment of advanced lung cancer. Conventional cancer drugs typically act directly on tumor cells, exerting cytotoxic effects. ICIs, on the other hand, block the interaction between inhibitory receptors expressed on T cells and their respective ligands, thus disrupting the immune checkpoint pathways within the tumor microenvironment.^[3,4]^ This further enhances the function of tumor-specific T cells, bolstering the endogenous antitumor immune response and resulting in antitumor effects.^[5,6]^

Previous studies have widely indicated that PD-1/PD-L1 inhibitors provide long-term survival benefits for lung cancer patients.^[7,8]^ However, not all patients benefit from these treatments. It has been reported that the cumulative effective rate of immunotherapy is <30% in lung cancer,^[9]^ and the side effects of these drugs are also a point of concern. Therefore, it is crucial to identify which patients can benefit from immunotherapy. Currently, PD-L1 expression, tumor mutational burden, and microsatellite instability-high (MSI-H) are recognized predictive biomarkers of treatment efficacy.^[10]^ However, the heterogeneity of the tests used across studies and relative cutoffs limit their clinical application, especially the PD-L1 expression status. Therefore, exploring economical and convenient peripheral blood markers in clinical practice to predict the effectiveness of immunotherapy is highly necessary.

Prognostic Nutritional Index (PNI), originally proposed by Buzby, is a simple index obtained by combining serum albumin concentration and peripheral blood lymphocyte count.^[11]^ Initially, it was used to assess a patient’s nutritional status and surgical risk level. Subsequently, PNI has been found widespread applications in evaluating the immune and nutritional status of surgical patients.^[12,13]^ Multiple studies have indicated that PNI can effectively assess the prognosis of various cancer patients.^[14–16]^ In recent years, PNI has gained popularity in clinical research on various cancers. It not only plays a role in assessing the nutrition and immune status of cancer patients but also holds significant value in predicting the prognosis of cancer patients including the lung cancer.^[17,18]^ However, there is still no definitive consensus on the relationship between the long-term therapeutic benefits of ICIs and PNI in lung cancer patients.

Therefore, this meta-analysis aimed to identify the prognostic value of PNI in lung cancer patients who received the ICIs.

## 2. Materials and methods

This meta-analysis was conducted according to the Preferred Reporting Items for Systematic Review and Meta-Analyses 2020.^[19]^

### 2.1. Ethical statement

The authors are accountable for all aspects of the work in ensuring that questions related to the accuracy or integrity of any part of the work are appropriately investigated and resolved. All procedures performed in studies that involved human participants were in accordance with the ethical standards of the institutional and/or national research committee and with the 1964 Helsinki Declaration and its later amendments or comparable ethical standards.

### 2.2. Literature search strategy

The Medline, CNKI, EMBASE, and Web of Science databases were searched up to August 20, 2023 for available studies. Following terms were used: PD-1, PD-L1, CTLA-4, ICI, pulmonary, lung, cancer, carcinoma, tumor, neoplasm, PNI, survival, prognosis, and prognostic. More detailed strategy was as follows: (PD-1 OR PD-L1 OR CTLA-4 OR ICI) AND (pulmonary OR lung) AND (carcinoma OR tumor OR neoplasm OR cancer) AND (prognostic nutritional index OR PNI) AND (survival OR prognostic OR prognosis). All references in included studies were reviewed.

### 2.3. Inclusion criteria and exclusion criteria

Studies which met following criteria were included: (1) patients were diagnosed with primary lung cancer and received ICIs for at least 1 month; (2) PNI was calculated as 10 × serum albumin (g/dl) + 0.005 × total lymphocyte count (per mm^3^)^[20]^; (3) patients were divided into different groups based on value of PNI; (4) the progression-free survival (PFS) or (and) overall-survival (OS) were compared between the high and low PNI groups with the hazard ratios (HRs) and 95% confidence intervals (CIs); and (5) full texts were available.

Besides, studies met following criteria were excluded: (1) insufficient, duplicated or overlapped data; (2) editorials, animal trials, case reports, or letters.

### 2.4. Data collection

Information including the first author, publication year, country, sample size, pathological type, previous treatment history, combination of other treatment, ICI drug, cutoff value of PNI, endpoint, HR, and 95% CI was extracted from included studies.

### 2.5. Quality evaluation

Methodological quality was evaluated according to the Newcastle-Ottawa Scale score and studies with Newcastle-Ottawa Scale score > 5 were with high-quality.^[21]^

### 2.6. Statistical analysis

The heterogeneity among studies was evaluated by I^2^ statistics and the Q test. When significant heterogeneity was detected representing as I^2^ > 50% and/or *P* < .1, the random effects model was used; or the fixed effects model was applied.^[22]^ HRs and 95% CIs were combined to evaluate the association between PNI and PFS or (and) OS. Subgroup analysis based on pathological type (NSCLC vs SCLC), previous treatment history (first-line treatment) and combination of other treatment (yes vs no) was performed. Sensitivity analysis was conducted to detect sources of heterogeneity and assess stability of our results. Meanwhile, Begg funnel plot and Egger test were conducted to detect publication bias, and significant publication bias was defined as *P* < .05.^[23,24]^ The analyses were conducted by STATA (version 15.0) software.

## 3. Results

### 3.1. Literature selection

**Figure 1.**
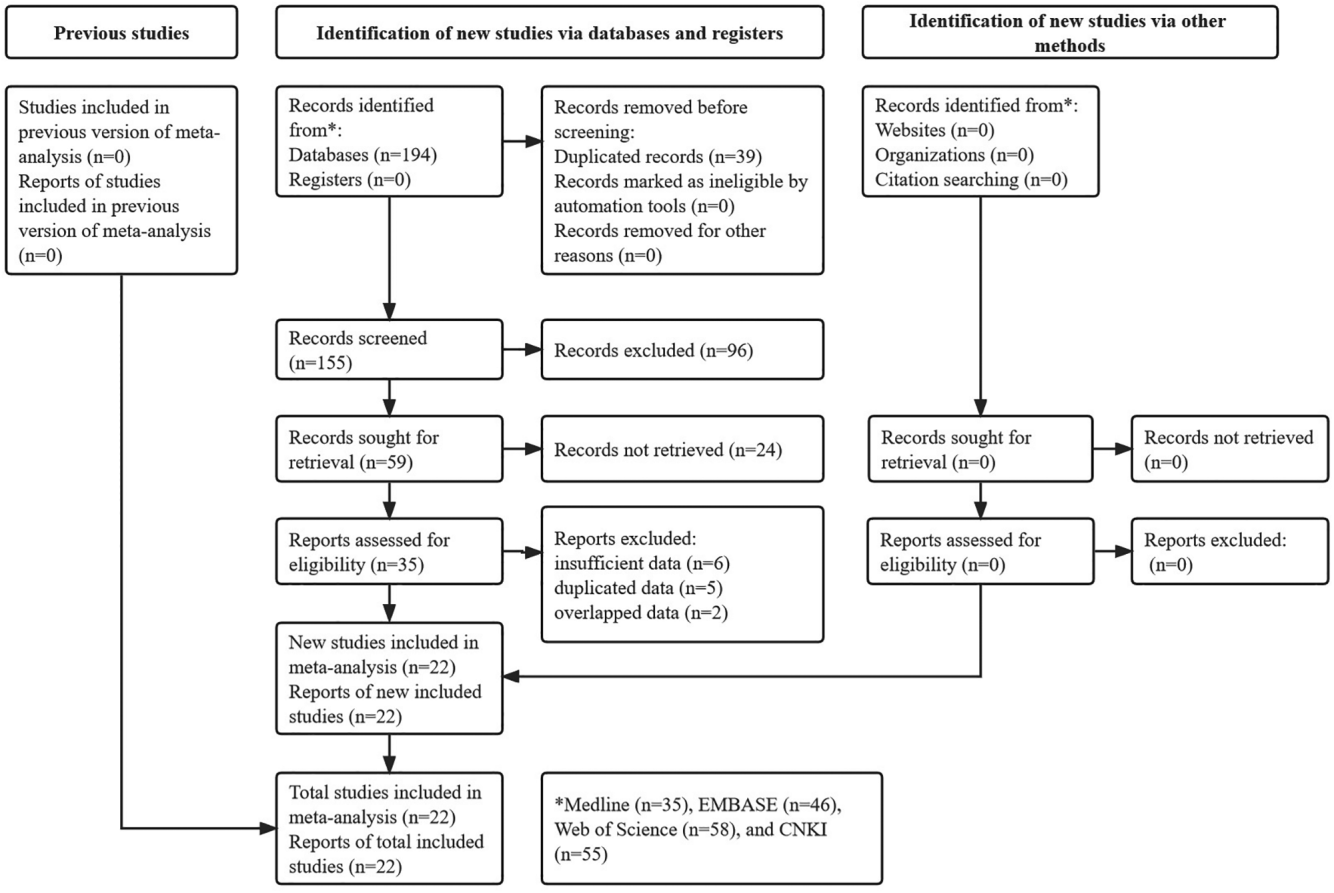
Prisma flow diagram of this meta-analysis.

As shown in Figure 1, 194 records were searched from the 4 databases. After screening titles, abstracts and full texts, 22 studies were eventually included.^[25–46]^

### 3.2. Basic characteristics of included studies

Detailed information of included studies was presented in Table 1. All included studies were retrospective with the overall sample size of 2550 cases. Most studies were from China or Japan and focused on NSCLC patients. The cutoff values of PNI ranged from 40 to 50.5. Besides, all studies were high-quality studies.

**Table 1:** Basic characteristics of included studies.

Author	Year	Country	Sample size	Pathological type	Treatment history	Combined treatment	ICI drugs	Cutoff value of PNI	Endpoint	NOS
Shoji^[25]^	2019	Japan	102	NSCLC	Mixed	No	Nivolumab, Pembrolizumab and Atezolizumab	45.5	PFS, OS	7
Matsubara^[26]^	2020	Japan	24	NSCLC	Mixed	No	Atezolizumab	48	PFS, OS	6
Peng^[27]^	2020	China	102	NSCLC	Mixed	No	Nivolumab, Toripalimab, Sintilimab and Pembrolizumab	45	PFS, OS	7
Ogura^[28]^	2020	Japan	34	NSCLC	No	Chemotherapy	Atezolizumab, Bevacizumab and Pembrolizumab	40	PFS, OS	6
Zaitsu^[29]^	2021	Japan	73	LC	Mixed	No	Nivolumab, Pembrolizumab and Atezolizumab	43	PFS, OS	7
Liu^[30]^	2021	China	123	NSCLC	Mixed	Mixed	Nivolumab, Pembrolizumab, Sintilimab, Camrelizumab and Toripalimab	46.05	PFS, OS	8
Qi^[32]^	2021	China	53	SCLC	No	Chemotherapy	Atezolizumab	48	OS	6
Shi^[33]^	2021	China	32	NSCLC	Mixed	No	NR	45	PFS, OS	7
Shi^[33]^	2021	China	71	NSCLC	Mixed	Chemotherapy	NR	45	PFS, OS	7
Luo^[34]^	2021	China	89	NSCLC	NR	Mixed	Nivolumab, Pembrolizumab, Tislelizumab, Camrelizumab and Toripalimab	41.15	PFS, OS	7
Qiu^[31]^	2021	China	105	NSCLC	Mixed	AAT/AAT + Chemotherapy	Pembrolizumab, Nivolumab, Toripalimab, Sintilimab, Camrelizumab and Atezolizumab	41.8	PFS, OS	7
Yi^[35]^	2021	China	121	NSCLC	Mixed	No	Pembrolizumab, Nivolumab, Toripalimab, Sintilimab, Camrelizumab and Tislelizumab	46.5	PFS	8
Fang^[36]^	2022	China	223	NSCLC	No	Chemotherapy	PD-1 inhibitor	50.5	PFS	7
Bolte^[37]^	2022	USA	92	NSCLC	NR	Chemotherapy	Pembrolizumab, Atezolizumab and Bevacizumab	41	OS	7
Stares^[39]^	2022	UK	219	NSCLC	No	No	Pembrolizumab	45	PFS, OS	8
Shijubou^[40]^	2022	Japan	38	NSCLC	No	No	Pembrolizumab	40	PFS	6
Tanaka^[41]^	2022	Japan	237	NSCLC	No	Chemotherapy	Pembrolizumab, Atezolizumab and Bevacizumab	40.35	PFS, OS	8
Chen^[38]^	2022	China	106	NSCLC	Mixed	Mixed	Sintilimab	45	PFS	6
Xu^[42]^	2022	China	124	NSCLC	Mixed	No	Pembrolizumab, Nivolumab, Toripalimab, Sintilimab, Camrelizumab and Tislelizumab	42.37	PFS, OS	7
Oku^[43]^	2023	Japan	91	NSCLC	No	No	Pembrolizumab	42.17	PFS, OS	8
Oku^[43]^	2023	Japan	127	NSCLC	No	Chemotherapy	Pembrolizumab and Atezolizumab	42.17	PFS, OS	8
Takeda^[44]^	2023	Japan	155	SCLC	No	Chemotherapy	Atezolizumab, Durvalumab	40	PFS, OS	7
Wu^[45]^	2023	China	61	SCLC	Mixed	No	NR	49.43	PFS, OS	8
Wu^[45]^	2023	China	79	SCLC	Mixed	Chemotherapy	NR	49.43	PFS, OS	8
Han^[46]^	2023	China	69	NSCLC	No	Chemotherapy	Pembrolizumab and Nivolumab	41.75	PFS	6

AAT = antiangiogenesis therapy, ICI = immune checkpoint inhibitor, LC = lung cancer, NOS = Newcastle-Ottawa Scale, NR = not reported, NSCLC = non-small cell lung cancer, OS = overall survival, PD-1 = programmed death 1, PFS = progression-free survival, PNI = prognostic nutritional index, SCLC = small cell lung cancer.

### 3.3. Association of PNI with PFS

**Figure 2.**
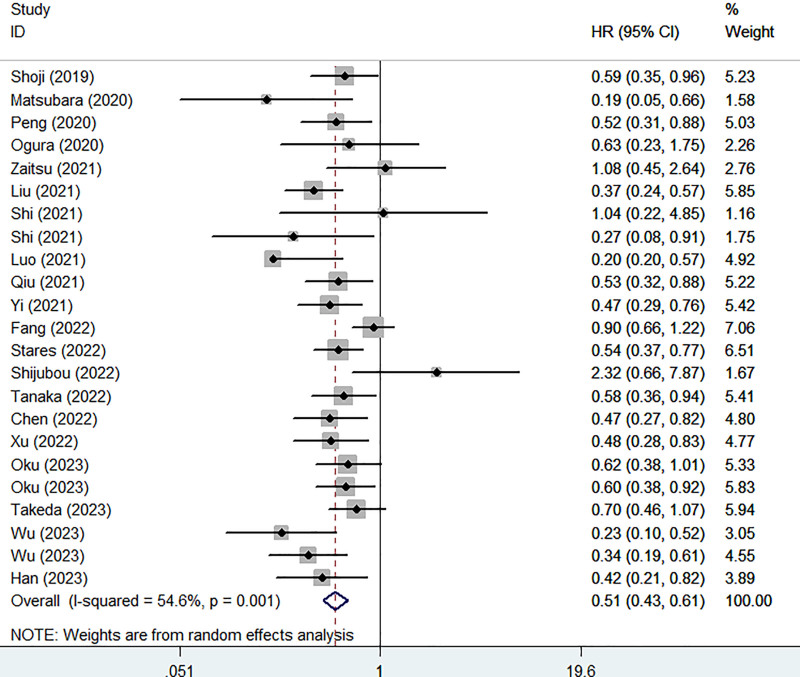
Association of prognostic nutritional index with progression-free survival in lung cancer patients receiving immune checkpoint inhibitors.

Twenty studies identified the association between PNI and PFS in lung cancer patients receiving ICIs. Pooled results manifested that lower PNI was significantly associated with worse PFS (HR = 0.51, 95% CI: 0.43–0.61, *P*<.001; I^2^ = 54.6%, *P* = .001) (Fig. 2). Furthermore, subgroup analysis demonstrated that PNI were related to PFS in lung cancer patients receiving ICIs despite of the pathological type (NSCLC: HR = 0.52, 95% CI: 0.43–0.62, *P*<.001; I^2^ = 52.8%, *P* = .004; SCLC: HR = 0.41, 95% CI: 0.21–0.79, *P* = .007; I^2^ = 73.2%, *P* = .024), previous treatment history (first-line treatment: HR = 0.67, 95% CI: 0.57–0.78, *P*<.001; I^2^ = 30.3%, *P* = .176), and combination of other treatment (No: HR = 0.54, 95% CI: 0.45–0.64, *P*<.001; I^2^ = 37.6%, *P* = .099; Yes: HR = 0.63, 95% CI: 0.53–0.74, *P*<.001; I^2^ = 40.8%, *P* = .096) (Table 2).

**Table 2:** Results of meta-analysis.

	Number of studies	Hazard ratio	95% confidence interval	*P* value	I^2^ (%)	*P* value
Progression-free survival	20	0.51	0.43–0.61	<.001	54.6	.001
Pathological type						
Non-small cell lung cancer	17	0.52	0.43–0.62	<.001	52.8	.004
Small cell lung cancer	2	0.41	0.21–0.79	.007	73.2	.024
First-line treatment						
Yes	8	0.67	0.57–0.78	<.001	30.3	.176
Combination of other treatment						
No	11	0.54	0.45–0.64	<.001	37.6	.099
Yes	9	0.63	0.53–0.74	<.001	40.8	.096
Overall survival	19	0.43	0.34–0.54	<.001	60.6	<.001
Type of lung cancer						
Non-small cell lung cancer	15	0.41	0.31–0.53	<.001	64.2	<.001
Small cell lung cancer	3	0.44	0.25–0.80	.007	54.4	.087
First-line treatment						
Yes	7	0.52	0.37–0.73	<.001	64.5	.006
Combination of other treatment						
No	10	0.43	0.33–0.56	<.001	26.2	.203
Yes	10	0.51	0.37–0.69	<.001	54.0	.021

### 3.4. Association of PNI with OS

**Figure 3.**
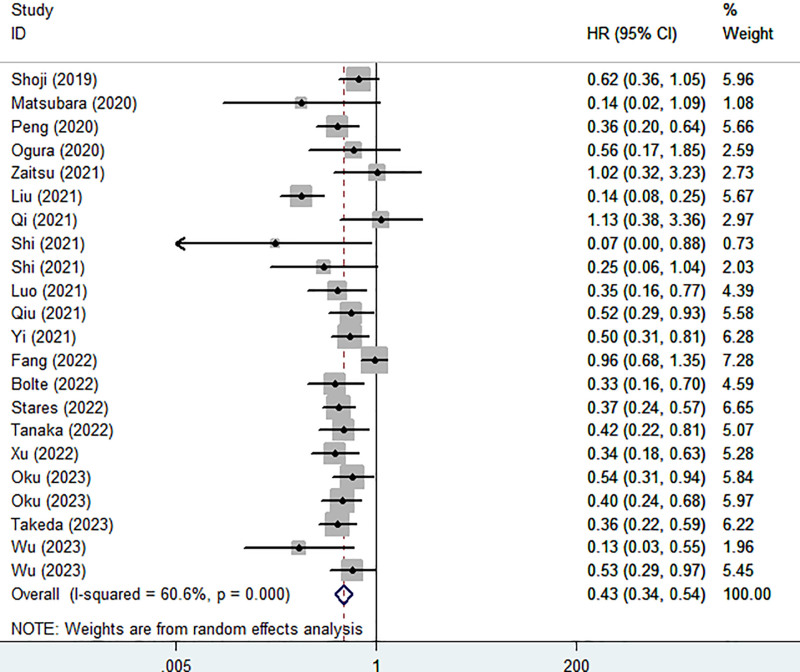
Association of prognostic nutritional index with overall survival in lung cancer patients receiving immune checkpoint inhibitors.

Besides, 19 studies explored the association between PNI and OS. Pooled results clarified that higher PNI was related to better OS (HR = 0.43, 95% CI: 0.34–0.54, *P*<.001; I^2^ = 60.6%, *P*<.001) (Fig. 3). Similarly, subgroup analysis stratified by the pathological type (NSCLC: HR = 0.41, *P*<.001; SCLC: HR = 0.44, *P* = .007), previous treatment history (first-line treatment: HR = 0.52, *P*<.001), and combination of other treatment (No: HR = 0.43, *P*<.001; Yes: HR = 0.51, *P*<.001) showed consistent results (Table 2).

### 3.5. Sensitivity analysis

**Figure 4.**
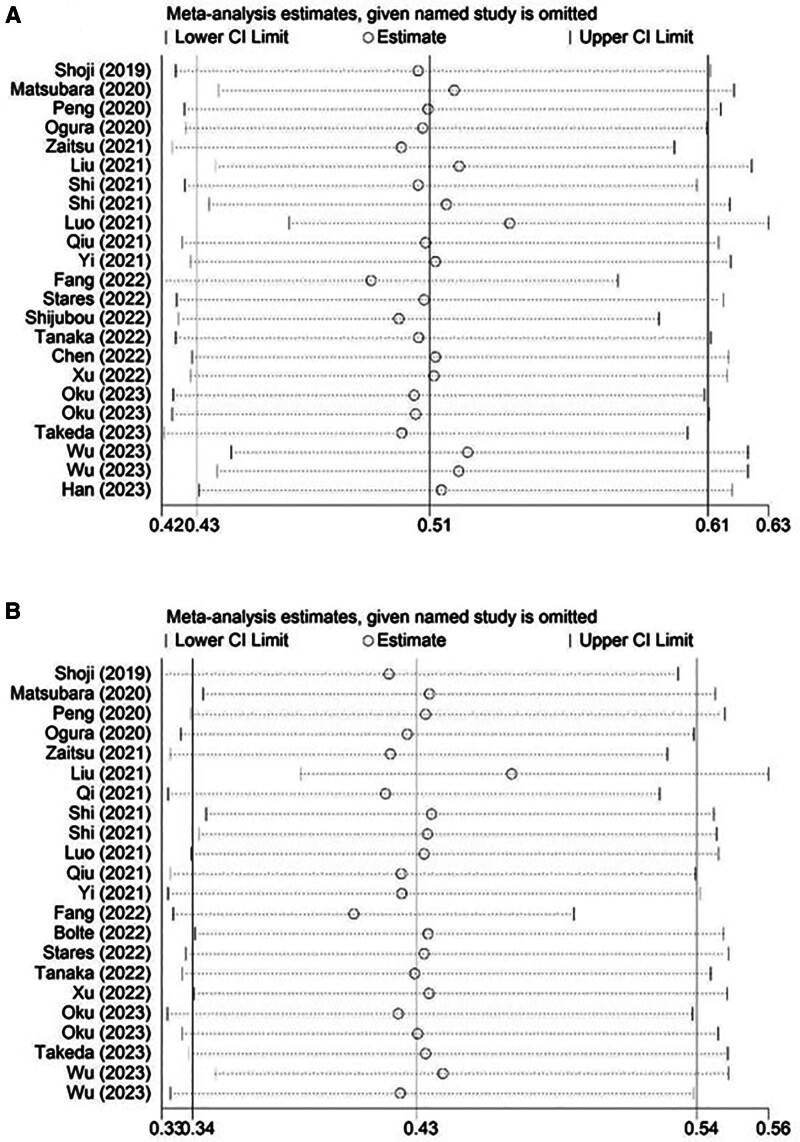
Sensitivity analysis for the association of prognostic nutritional index with progression-free survival (A) and overall survival (B) in lung cancer patients receiving immune checkpoint inhibitors.

Sensitivity analysis for the PFS (Fig. 4A) and OS (Fig. 4B) were both conducted, which showed that our results were stable and reliable and none of included studies caused an obvious impact on the pooled results.

### 3.6. Publication bias

**Figure 5.**
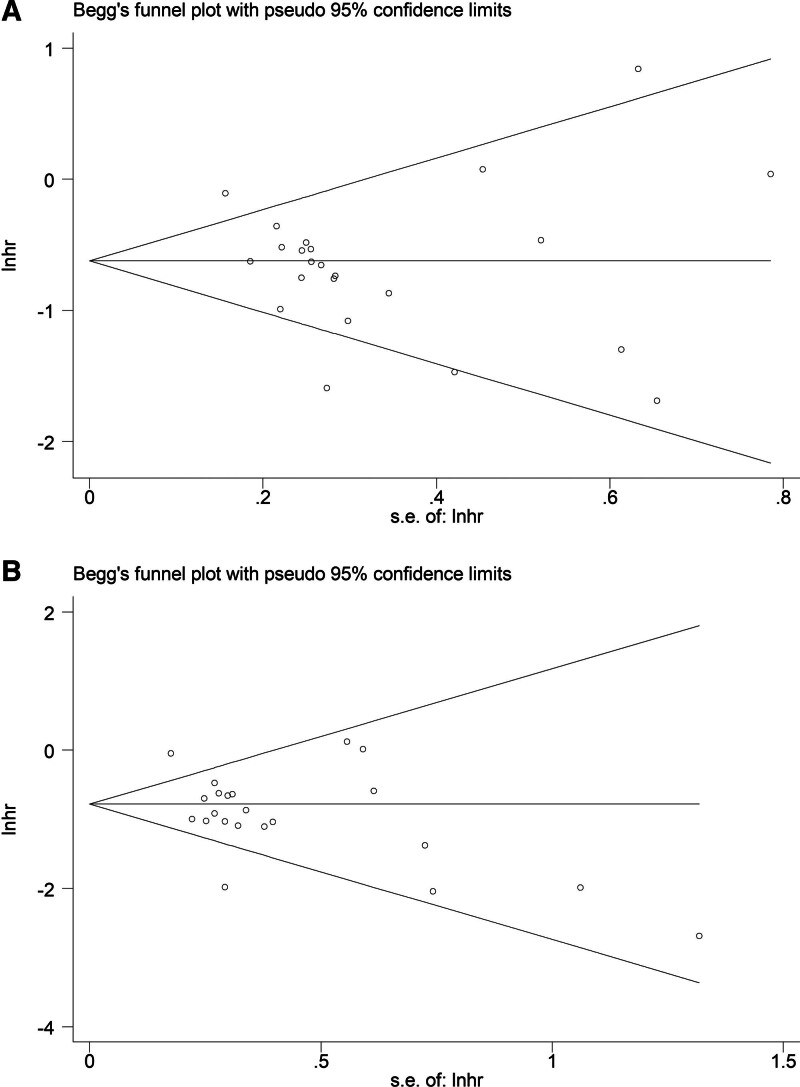
Begg funnel plots for the association of prognostic nutritional index with progression-free survival (A) and overall survival (B) in lung cancer patients receiving immune checkpoint inhibitors.

Begg funnel plots (Fig. 5A and B) and Egger tests (*P* = .334 and *P* = .103) both indicated that none significant publication bias existed in this meta-analysis.

## 4. Discussion

In this meta-analysis, a total of 22 studies with 2550 patients were included. Based on these available studies, it is suggested that PNI is significantly related to the long-term survival among lung cancer patients who receive the ICIs and patients with a lower PNI are more likely to experience worse prognosis.

The expression level of PD-L1 in tumor cells is currently the most commonly used predictive marker for the efficacy of immunotherapy. Results from 2 major clinical studies, Checkmate 057^[47]^ and Keynote-010,^[48]^ both suggest that patients with high PD-L1 expression in tumor tissues benefit more from anti-PD-1/PD-L1 immunotherapy. However, the Checkmate 017 study showed cases where PD-L1-negative patients responded well to immunotherapy.^[49]^ Moreover, the reliability of PD-L1 expression as a predictive marker for immunotherapy efficacy is limited due to the inconsistencies in results caused by factors such as differences in the brand of antibodies used, testing techniques, testing environmental conditions, and the varying cutoff values used to determine PD-L1 positivity. Tumor mutational burden has been another research focus as a predictive marker for cancer immunotherapy efficacy. However, it remains a reference marker due to factors like tumor heterogeneity and varying thresholds, and further extensive clinical research is needed to explore its effectiveness. In May 2017, the U.S. FDA approved Pembrolizumab for the treatment of advanced or metastatic solid tumors with MSI-H or DNA Mismatch Repair Deficiency. This brought a new wave of interest in immunotherapy biomarkers. Nonetheless, it was reported at ASCO in 2016 that the incidence of MSI-H in lung cancer was only 0.4% to 0.8%. Therefore, the role of MSI-H or DNA Mismatch Repair Deficiency in predicting the efficacy of immunotherapy in lung cancer requires further investigation. While the aforementioned predictive markers for immunotherapy efficacy are generally accepted, they all face issues related to the complexity and high cost of the testing process. Thus, the search for economical and convenient predictive markers for therapeutic efficacy is of great significance.

Actually, the prognostic value of PNI in some cancers patients treated with ICIs has been manifested by meta-analyses. Xu et al included 23 studies with 2386 cancer patients and indicated that low PNI was related to poor PFS (HR = 1.75, *P*<.001) and OS (HR = 2.26, *P*<.001) and low objective response rate [odds ratio (OR) = 0.47, *P*<.001] and disease control rate (OR = 0.43, *P*<.001).^[50]^ Besides, Zhang et al included 17 studies with 2883 participants and demonstrated that patients with PNI showed longer PFS (HR = 0.74, *P*<.001) and OS (HR = 0.53, *P*<.001) and higher objective response rate (OR = 1.622, *P*<.004) and disease control rate (OR = 1.846, *P*<.001) among gastrointestinal cancer patients treated with ICIs.^[51]^ As for lung cancer, in the meta-analysis by Xia, 13 studies with 1119 advanced NSCLC patients were included and the significant association between lower PNI and worse PFS (HR = 1.84, *P*<.001) and OS (HR = 2.68, *P*<.001) was observed.^[52]^ However, their subgroup analysis manifested that PNI did not play a role in predicting the survival among patients with the combination of chemotherapy (HR = 1.67, *P* = .19) or with previous treatment (HR = 1.61, *P* = .20). Our meta-analysis included much more patients and indicated that above factors did not show a significant impact on the prognostic role of PNI in lung cancer patients receiving ICIs.

Based on relevant reports, it is suggested that there is a special association between PNI and the efficacy and toxicity of ICIs therapy. Albumin levels reflect the body’s nutritional status and immune function.^[53,54]^ It has been well clarified that nutritional status is closely related to the efficacy of ICIs therapy. Malnutrition may lead to chronic inflammation, and there is a connection between inflammation and immune suppression^[54]^. Elevated levels of inflammation may potentially diminish the effectiveness of immunotherapy. Optimal nutritional status contributes to maintaining the functionality of cytotoxic T cells, which are crucial for identifying and destroying cancer cells^[53]^. Adequate nutrition can provide sufficient antioxidants, aiding in reducing oxidative stress, which is essential for sustaining the health and vitality of immune cells^[54]^. Besides, low albumin levels have been reported to be associated with immune-related adverse events, including toxic reactions that may affect multiple organ systems.^[45,53]^ These reactions can increase inflammation in the body, potentially affecting the synthesis and secretion of albumin. Additionally, albumin is primarily synthesized in the liver, so it is closely related to liver function. Some ICI drugs may cause liver dysfunction, leading to a decrease in albumin levels.^[55,56]^ In addition, a low lymphocyte count may also be associated with immunotherapy toxicity. Lymphocytes are a key component of the immune system responsible for identifying and attacking foreign substances, including tumor cells. Therefore, the success of immunotherapy often relies on a robust immune response. If a patient has a low lymphocyte count, it may impact the normal function of the immune system and increase the risk of toxicity associated with immunotherapy.^[57]^ Among patients undergoing immunotherapy, some common toxic reactions are related to the activity of the immune system, including immune-related thyroiditis, skin inflammation, pneumonia, and others.^[58–60]^ A low level of lymphocytes may lead to insufficient response to these reactions, as lymphocytes play a crucial role in regulating and executing immune responses.^[58,59]^

Furthermore, there is also a specific association between lung cancer and PNI. Chronic obstructive pulmonary disease is a common comorbidity in lung cancer patients. In chronic obstructive pulmonary disease patients, due to chronic inflammation and airway obstruction, the immune system is often activated, leading to an increase in lymphocyte levels.^[61]^ The mechanisms involved include chronic inflammation, airway obstruction, and oxidative stress.^[61]^ Additionally, chronic obstructive pulmonary disease patients are more prone to developing hypoproteinemia.^[62]^ Meanwhile, Smoking is the most significant risk factor for lung cancer. Smoking typically leads to an increase in lymphocyte levels.^[63]^ The inflammatory response triggered by smoking may activate the systemic immune system, including an increase in lymphocytes.^[63]^ Chemical substances in tobacco smoke may induce oxidative stress, representing an immune response against harmful substances and resulting in an elevation of lymphocytes.^[64]^ Smoking may also elevate the risk of respiratory infections, prompting an immune response, including heightened activity of lymphocytes.^[64]^

Therefore, we believe it is essential to further elucidate the correlation between the PNI and the prognosis of lung cancer patients receiving ICIs. Based on the aforementioned reasons, this phenomenon may possess a certain specificity in lung cancer patients receiving ICI treatment.

Furthermore, the relationship between changes of PNI during the immunotherapy and therapeutic effects could be investigated in future relevant studies. More specific optimal cutoff values of PNI predicting therapeutic effects in lung cancer are needed, which might contribute to the application of PNI in clinics. Besides, it is also valuable to explore the impact of improvement of PNI in improving the prognosis in lung cancer patients treated with ICIs.

There are several limitations in the meta-analysis. First, the sample size is relatively small and all included studies are retrospective. Secondly, we could not conduct more detailed analysis based on other important parameters such as the drugs and age because original data are unobtained. Thirdly, short-term outcomes of ICIs such as the objective response rate and disease control rate were not observed in this meta-analysis. Fourthly, most included studies are from Asian counties and the sample size of some included studies is relatively small, which might cause some bias.

## 5. Conclusion

PNI is significantly associated with long-term survival among lung cancer receiving ICIs and patients with a lower PNI experience worse prognosis.

## Author contributions

**Conceptualization:** Lei Wang, Mei Yang.

**Data curation:** Lei Wang, Xingxia Long, Ying Zhu.

**Formal analysis:** Xingxia Long, Ying Zhu.

**Investigation:** Lei Wang, Xingxia Long.

**Methodology:** Ying Zhu.

**Resources:** Ying Zhu.

**Software:** Lei Wang, Xingxia Long, Ailin Luo.

**Supervision:** Mei Yang.

**Validation:** Ailin Luo.

**Visualization:** Ailin Luo.

**Writing – original draft:** Lei Wang.

**Writing – review & editing:** Ailin Luo, Mei Yang.
